# Lockdown in a disneyfied city: Kraków Old Town and the first wave of the Covid-19 pandemic

**DOI:** 10.1057/s41289-021-00175-5

**Published:** 2021-10-04

**Authors:** Anna Porębska, Krzysztof Barnaś, Bartosz Dendura, Olga Kania, Marta Łukasik, Aleksandra Rogulska, Ernestyna Szpakowska-Loranc, Miłosz Zieliński

**Affiliations:** Faculty of Architecture, Cracow University of Technology, 24 Warszawska Street, 31-155, Kraków, Poland

**Keywords:** Cultural heritage sites, Disneyfication, Post-2020 planning, Kraków, Poland

## Abstract

This paper presents the geography of the historic central district of Kraków, Poland before, during and after the first wave of the 2020 pandemic. It describes how the disneyfied main part of the UNESCO heritage site of universal values turned into a ghost town as functional changes were turning into physical ones amid restrictions. From the results of pre-pandemic processes (that, as we argue, turned the city into its disneyfied version), to the lockdown (that later revealed itself to be but the first one in a row), to the post-lockdown recovery, these changes are presented in modified figure-ground diagrams with accessibility being defined by both tangible and intangible properties. The results are set against the background of the city’s current policies regarding economic recovery, mobility and accessibility to urban green areas. As an attempt to address the present vulnerability of the once resilient historic city centres—of which Kraków Old Town is a luminous example—this paper tends to be a voice in the debate on the post-2020 planning and the strategies we will need to face the subsequent waves of this, or other, pandemics as well as consequences of climate change.

## Introduction

### The city and the plague

The question about the right to the city, asked long before Lefebvre’s seminal work, has taken on a new dimension during the Covid-19 pandemic. While forcing over half of the planet’s population to stay at home has slightly relieved urban ecosystems (cf. Braga et al. 2020, among others), it has starkly reminded us of the role that public places and spaces played in everyday life. And yet, while the international academic community has done its best to tackle the associated medical, social and economic problems, the role that the built environment has played during the pandemic, and whether this experience may help us survive future challenges, remains largely unspecified.

Publications regarding connections between the built environment and infectious diseases from historical perspectives (Engelmann et al. 2018) and the erosion of our relationship with spaces, along with its consequences (Porębska 2016, among others), existed before 2020. A set of planning measures that cities can adopt to alleviate the consequences of an outbreak was proposed years ago by Matthew and McDonald (2006), stressing interdisciplinary cooperation in pandemic-focused disaster planning. Differences in epidemic development models in urban settings were studied by Wolf (2016), who explored it from three different perspectives: health, nature and networks, yet provided little in the way of specific guidelines or courses of action.

Among the first to address the situation was Acuto (2020), who closely looked at urbanism and examined how different forms of development and development-related phenomena created favourable conditions for epidemics. Acuto noted that the Covid-19 pandemic exposed existing issues such as overcrowding, unmanageability of suburban sprawl, increasing prevalence of informal settlements and pervasive inequality, but did not delve deeper into these problems. Honey-Roses et al. (2020) explored how central and municipal authorities in many countries seized control over public spaces and used them as corridors for emergency services, essentially reducing them to their circulatory role. Discussions have also started on how the pandemic has affected urban and political life in the context of resilience and how some of these changes may persist (Fistola and Borri 2020; Van Assche et al. 2020), as well as how data collection can improve future epidemic response planning (Campagna 2020).

Among the first conclusions from the early 2020 pandemic reality, those aimed at eliminating inclusive urban spaces due to posing an epidemic risk prevailed (Landman 2020). However, what these conclusions did not offer was a long-term perspective—whether past or future oriented. Neither did papers by Noworól (2020) who presented the Covid-19 pandemic as a stimulator of antispatiality processes and Lorens (2020) who characterized a new model of a post-pandemic city with a low-density structure that satisfies essential needs in the immediate vicinity of housing areas. It was not until more than a year after the pandemic outbreak, when the academic community, social organizations and municipal authorities—UN Habitat included—started to formulate rules for creating a user-friendly and safe urban space in Covid-19 times and the post-Covid new normality.

Our study addresses imperfections of the built environment the quality of which was sacrificed on the grounds of constant growth and track the patterns typical for temporarily deserted monofunctional areas, in particular those dominated by offices and administration buildings in tourist-oriented city centres. In some cases, like Venice, whose ecosystem flourished amid the lack of tourist (Braga et al. 2020, among others), the change resulting from the sudden lack of tourists was positive. However, there are also some negative consequences, the historic city centre of Kraków, Poland being among the examples.

We present how, during the first wave of the Covid-19 pandemic, in early spring 2020, the central part of Kraków revealed itself a deserted and high-maintenance scenography rather than the vibrant core it used to be. We also discuss what impact this experience should have on the city’s strategies regarding the local economy, mobility and green networks. Some patterns revealed in the study can be considered universal for other tourist-reliant city centres in these and similar circumstances. Furthermore, a survey held as part of this research (Porębska and Dendura 2020) provides background for a bottom-up alternative to the top-down strategies regarding Covid-related countermeasures.

### Disneyfied city: beyond gentrification

Disneyfication, also called disneyization or disneylandization, is a widely recognized phenomenon, applicable to space as well as entire societies and behaviours (Zukin 1996; Bryman 1999). Interesting insights were later provided by Sequera and Nofre (2018) who directly associated it with gentrification. It is often seen as synonymous with, or closely tied to, overtourism—whose effects on Kraków, Poland were discussed by Plichta (2019) and Lorens (2006), among others. Some have argued that areas placed on the UNESCO World Heritage List can be more prone to this effect (Cuccia et al. 2016).

**Fig. 1:**
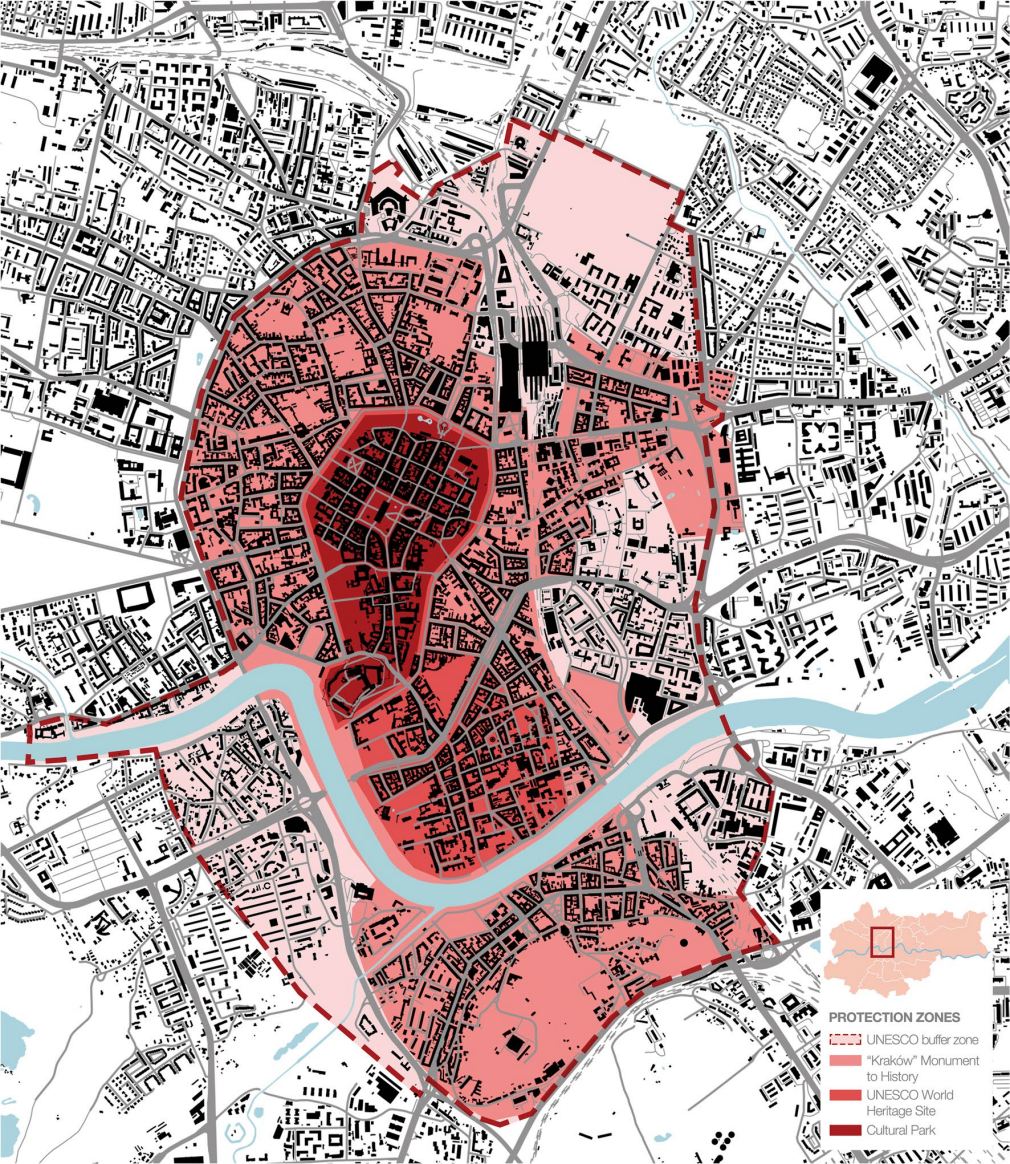
Kraków city centre and its conservation strategies

**Fig. 2:**
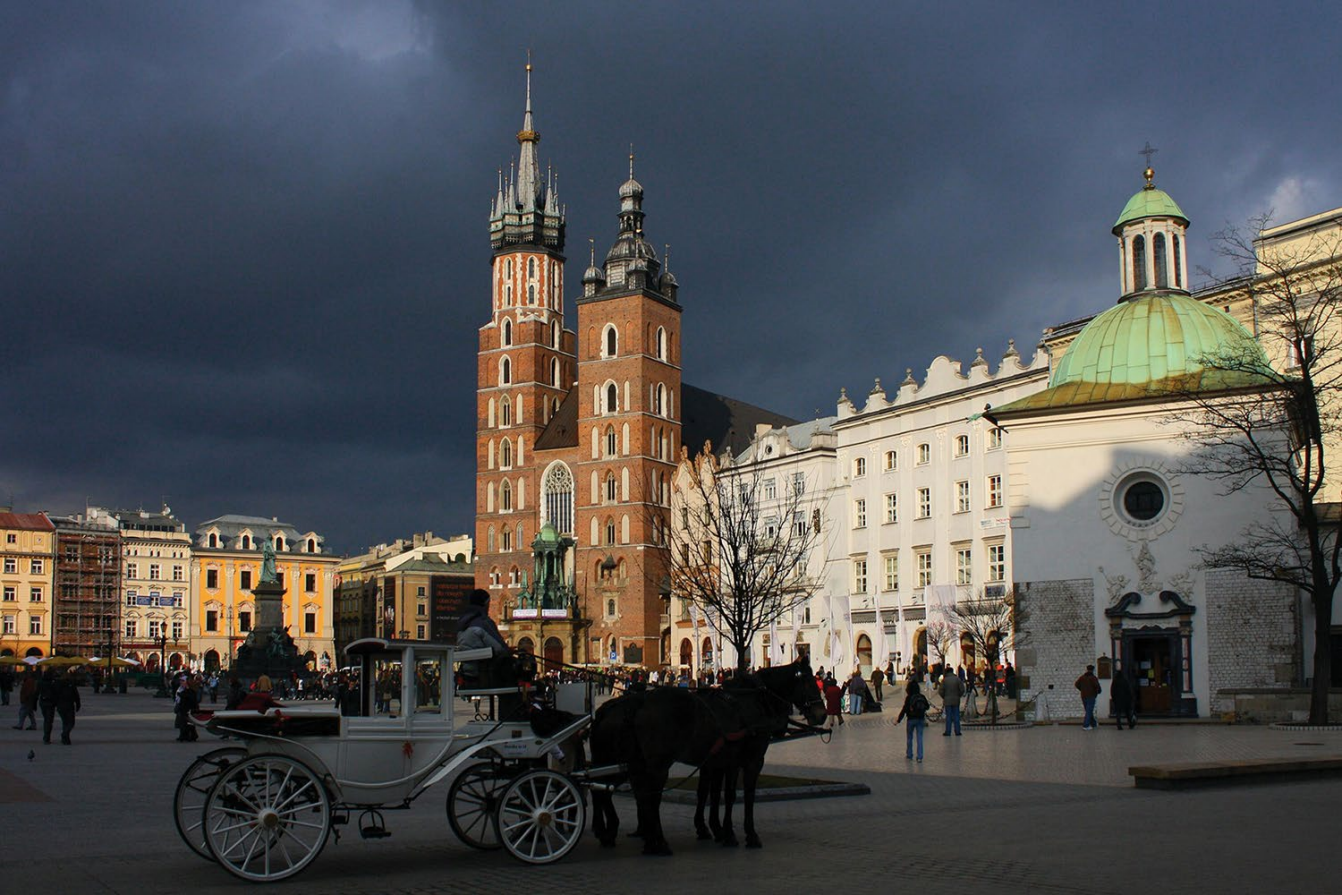
Kraków, Market Square (Rynek) with St. Mary and St. Adalbert Churches. Photo by Ludwig Schneider / Wikimedia Commons (2009) CC-BY

**Fig. 3:**
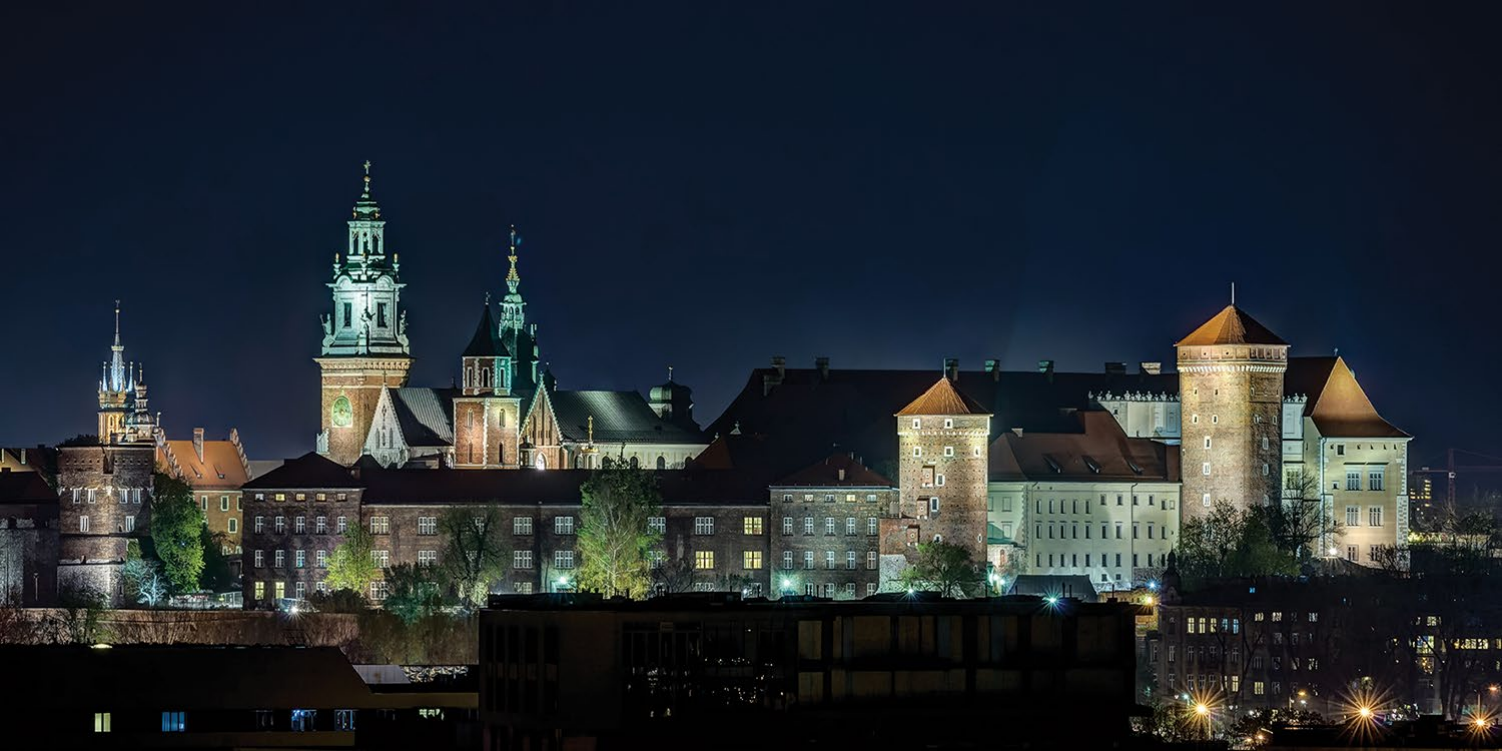
Kraków, Wawel Hill. Photo by Jar.ciurus / Wikimedia Commons (2012) CC-BY

The historic centre of Kraków, Poland was among the first eight sites chosen for the original 1978 UNESCO World Heritage Sites List (Fig. 1). Kraków Old Town (Cracovia) is famous for its urban grid with the Main Market Square as well as for its fairy-like architecture (Fig. 2); Wawel Hill—for its monumental silhouette dominating the city skyline, Gothic cathedral and Renaissance courtyard (Fig. 3); Kazimierz—for its Jewish quarter.

Before the 2020 pandemic put everything on hold, Kraków was among one of the most popular tourist destinations in Europe. Compact and relatively affordable, it specialized in short-term tourism. It is a profile typical for theme parks and is associated with merchandising, hybrid consumption and foodification as a consequent result. Foodification is a phenomenon of turning historic centres into a food-dominant retail space whose effects on Florence and Italy have been explored by Loda et al. (2020). It can easily be argued that Kraków’s historic centre, in particular the Jewish quarter—Kazimierz—have been heavily foodified over the past decade, although it has yet to attract academic interest as a case. Kraków’s nightlife can also be seen as disneyfied, when analysed following Nofre and Martins’ approach (2017): with the night becoming a time–space for youth relationships and diversions, mostly dominated by vast numbers of university students.

**Fig. 4:**
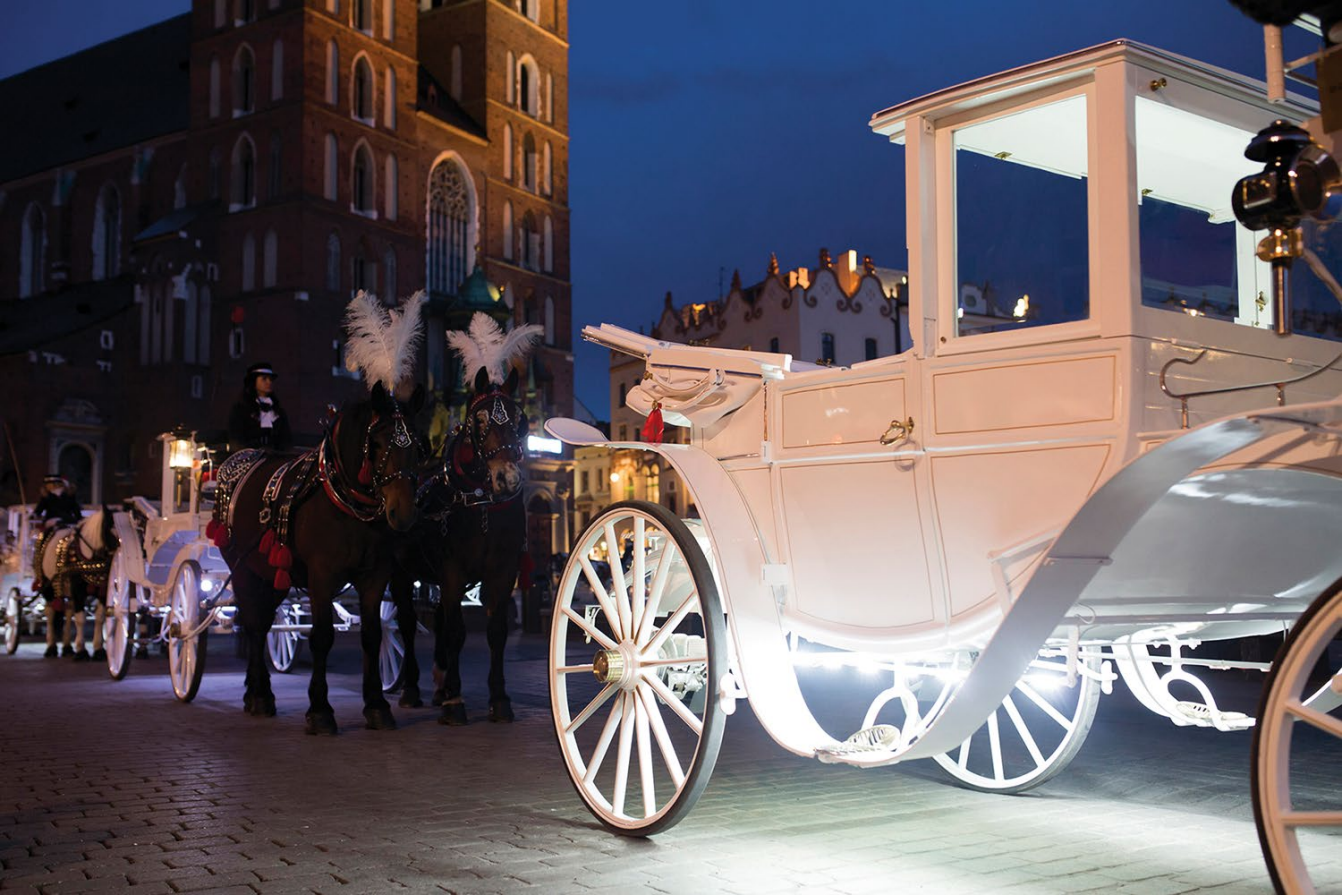
As recently as in the second part of the twentieth century, horse carriages were used in Kraków as an alternative transport mode. Along with mass tourism, traditional one-horse carriages were replaced by fancy, fairytale-like landaus. Photo by Andrzej Banaś / Polska the Times (2016) ©

Over the last decades, Kraków Old Town turned into its tourist-friendly, sanitized and beautified version, with e.g. fairytale-like landaus (Figs. 2, 4) replacing traditional carriages. Plagued by similar problems as those of other heavily touristic European cities, such as Florence or Venice (Seraphin et al. 2018), Kraków can serve as a representative case for the study of disneyfication and overtourism, in addition to how these phenomena affect cities during the Covid-19 pandemic. It can also be considered a post-Covid-19 ‘ghost town’ as defined by Florida and Cooke (2021).

**Fig. 5:**
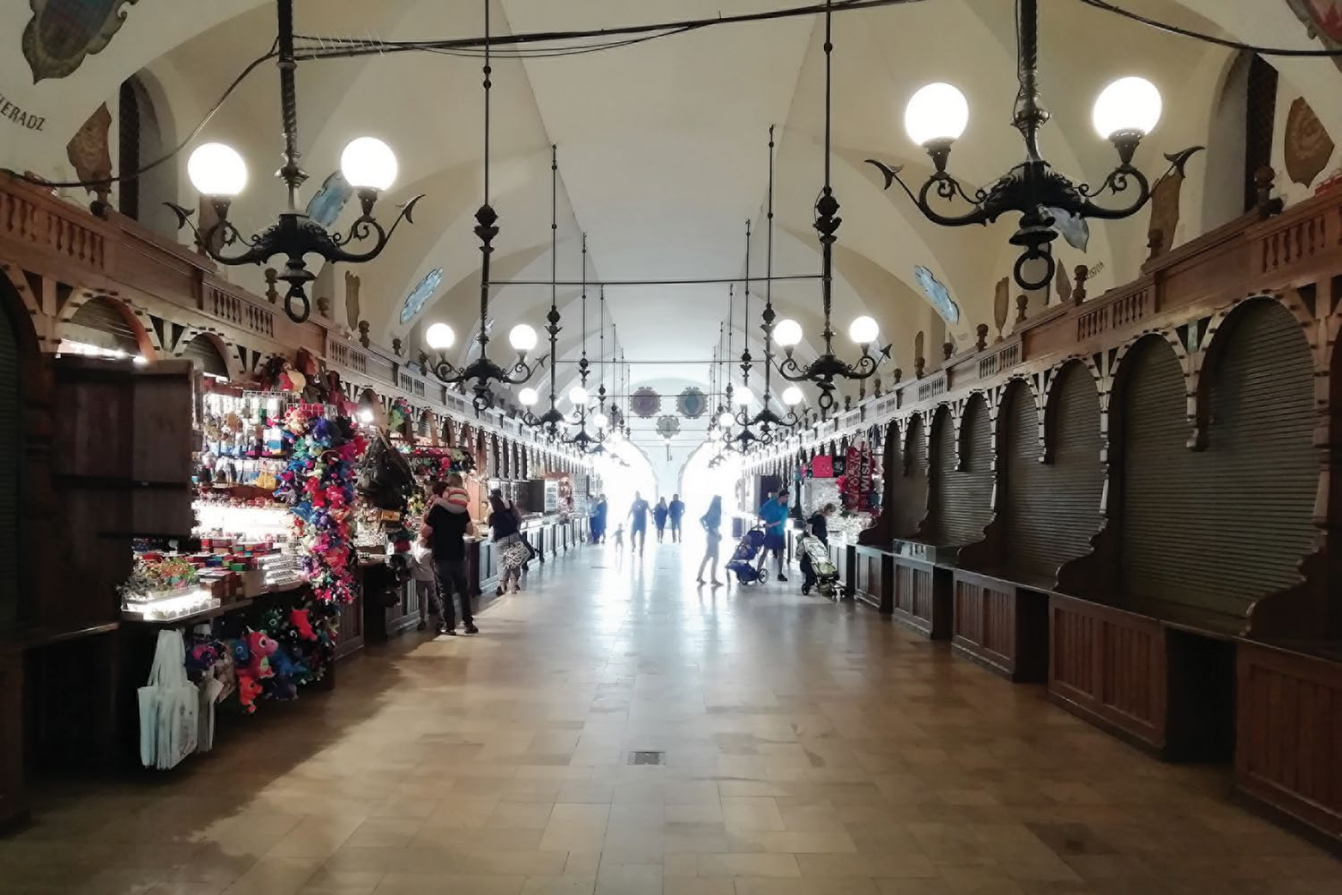
Stalls in the internal gallery of Sukiennice (Cloth Hall), July 2020. The central feature of the Main Market Square with its current form dating back to Renaissance is one of the city’s most recognizable icons. Photo by Aleksandra Rogulska

The detailed field survey presented in this paper focused on the oldest part of the city, i.e. the Old Town with Wawel Hill, for a number of reasons. First, they constitute the very foundation for the local identity—for nearly a million residents of the agglomeration, the word ‘city’ refers exclusively to this area of less than 0.9 km^2^. Other districts, historic and modern alike, are referred to by their proper names. Second, framed by Wawel Hill with the Royal Castle and the green belt of Planty Park that replaced the mediaeval city walls, it is the most recognizable part of Kraków, central both literally and figuratively. Third, it is placed under all forms of conservation currently in effect within the area of the historic layout (Fig. 1). Finally, the area meets all of the criteria for disneyfication as defined by Lorens (2006): it is a historic space, which has become the object of historicizing stylizations (Figs. 2, 3, 4, 5) subsequently encouraged by the provisions of the Cultural Park (cf. Resolution of the Kraków City Council of 13 February 2019 No. VII/128/19 on accepting the Resolution No. CXV/1547/10 on the Old Town Cultural Park), it is a functional structure adapted largely to mass tourism, and its depopulation goes beyond ‘ordinary’ gentrification.

As for the area’s gentrification, it is not that it lacks low- and average-income residents, which is typical for gentrified areas, but that it lacks residents altogether. Officially, according to the last census of 2014, the area encircled by Planty Park was inhabited by approximately 2500 people—a quarter of its fourteenth-century population. Since then, its depopulation has continued. Furthermore, due to a specific and complicated post-war and post-communist ownership structure that was not solved until recently (often in semi-transparent or inconclusive procedures) combined with the arrival of mass tourism, many buildings in the city centre were turned directly into commercial buildings, hotels, aparthotels, etc., so the centre somewhat skipped the phase of gentrification without ever being dwelled by wealthy locals.

### Lockdown in a disneyfied city: methods

To present how the Covid-19 lockdown had affected the city, we performed a field survey of the Kraków Old Town, examining how local accessibility of public spaces and buildings changed.

**Fig. 6:**
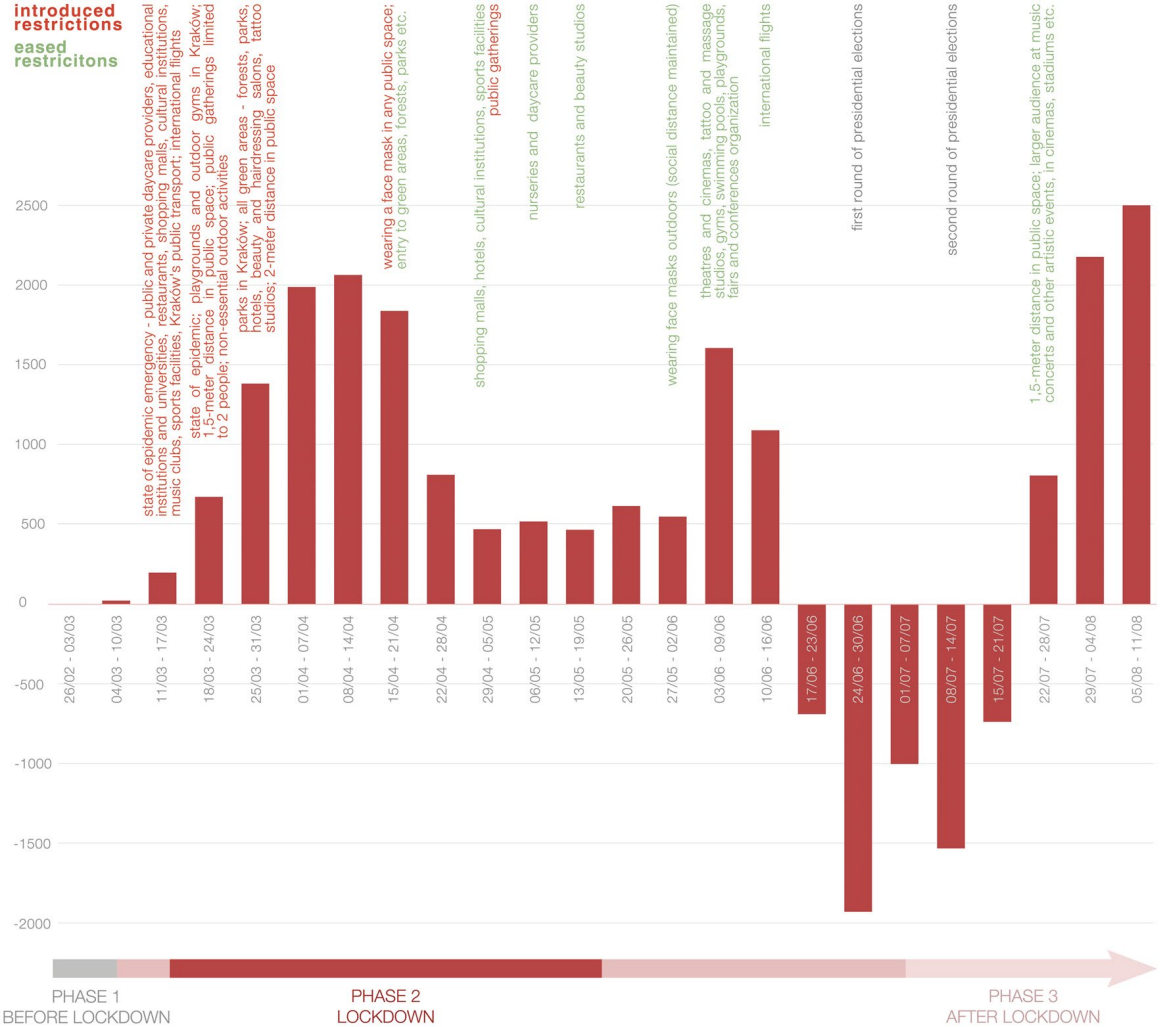
Chronology of active cases and strategies applied during the first wave of the Covid-19 pandemic in Poland. Data based on official communication and statistics published daily on gis.gov.pl

In Poland, while insecurity and compulsive purchasing of non-perishables were omnipresent amid dramatic news coming from Italy and Spain, the first days of March 2020 still resembled everyday life. It was not long, however, for the country to close the borders as well as cultural institutions like cinemas, theatres, museums, and operas, and suspend flights as well as school and university classes, immediately after the first case was confirmed. It can be agreed upon that the lockdown period in Poland started between 13 and 20 March 2020, when first a state of epidemic threat, and later an epidemic state was declared nationwide. Such a state is not to be confused with states of emergency as defined by the Polish Constitution (Chapter XI, Article 228) that have not been announced either during the first or the second wave of the pandemic. Gatherings, in excess of 2 people, were banned, social distancing (2 m, subsequently reduced to 1.5 m) mandated, mobility limited to a minimum, and the maximum capacity for public transport reduced to half the number of seats. Restaurants, bars, and cafes were closed, only permitting takeaway and delivery services, as well as stores in shopping centres, except for those providing access to essential needs and services. The indicative period of lockdown is given as the time between 31 March, when restrictions on movement for minors were imposed, the public administration switched to working remotely, parks, forests and boulevards were closed, and 20 April, when they were reopened effective immediately. The chronology of active cases by weeks and preventative measures implemented by the Polish government are summarized in Fig. 6.

**Fig. 7:**
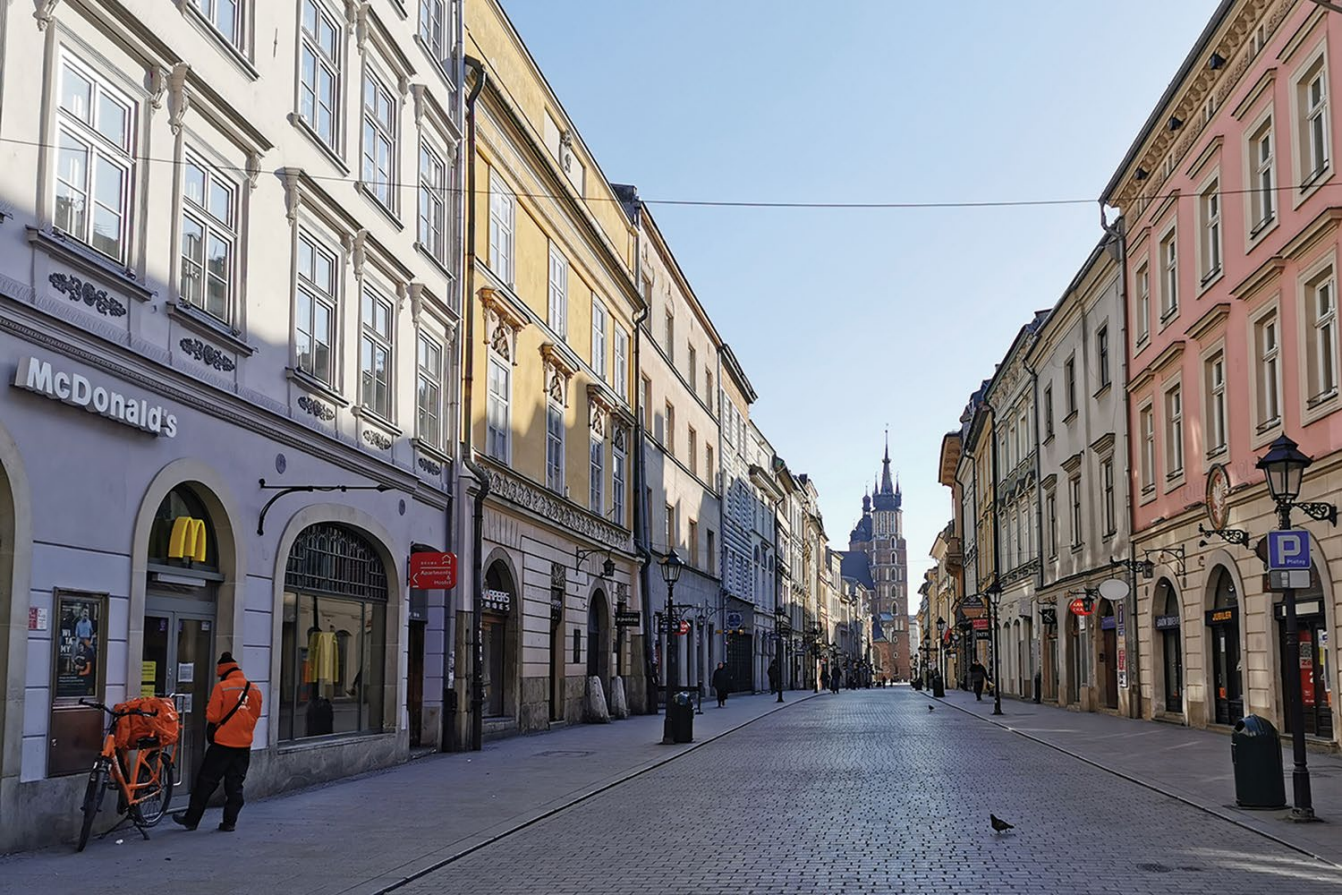
Floriańska Street, the usually crowded northern part of the Royal Route, with the St. Mary’s Basilica seen in the distance. Sunny afternoon in April 2020. Photo by Miłosz Zieliński

**Fig. 8:**
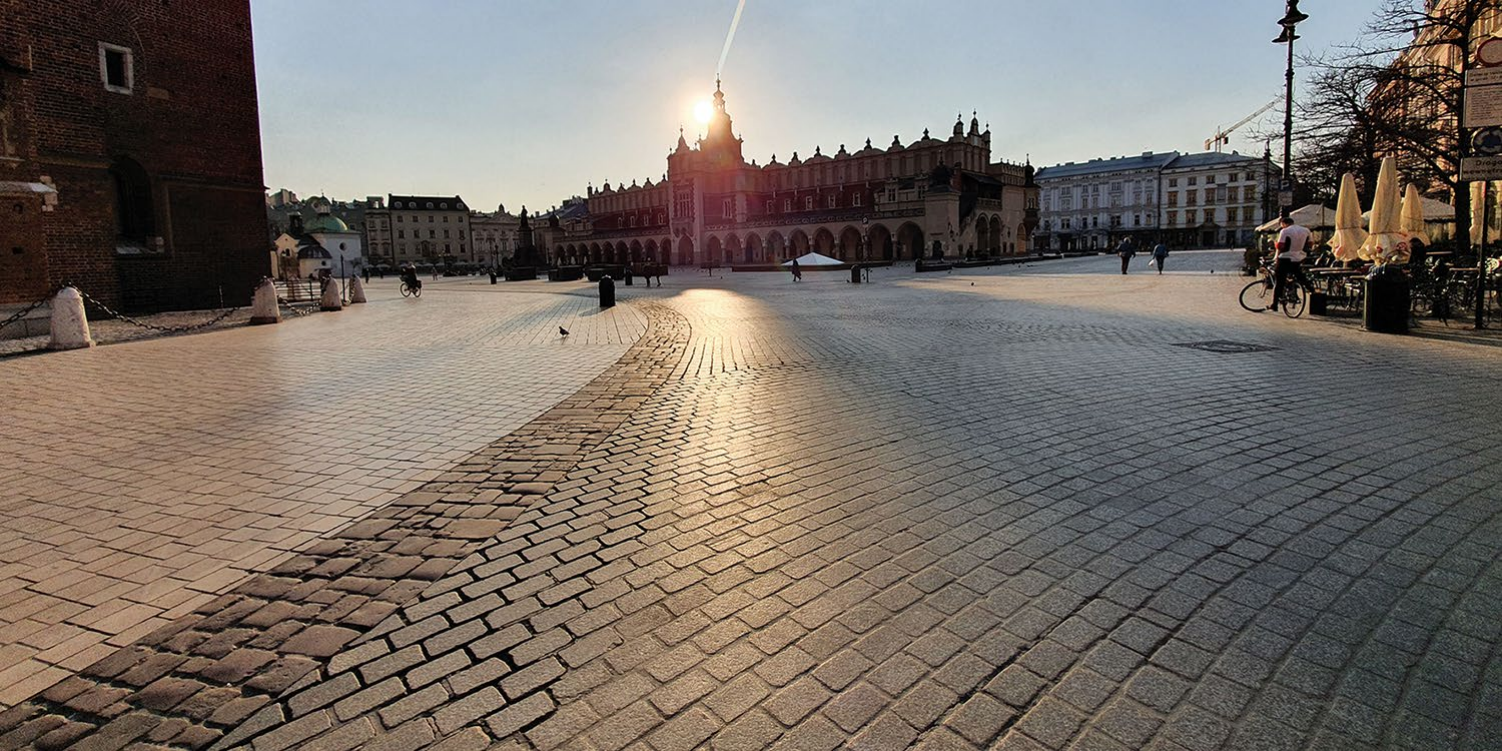
Main Square, 17 March 2020, 5 PM (238 confirmed cases, 61 that day). Photo by Bartosz Dendura

**Fig. 9:**
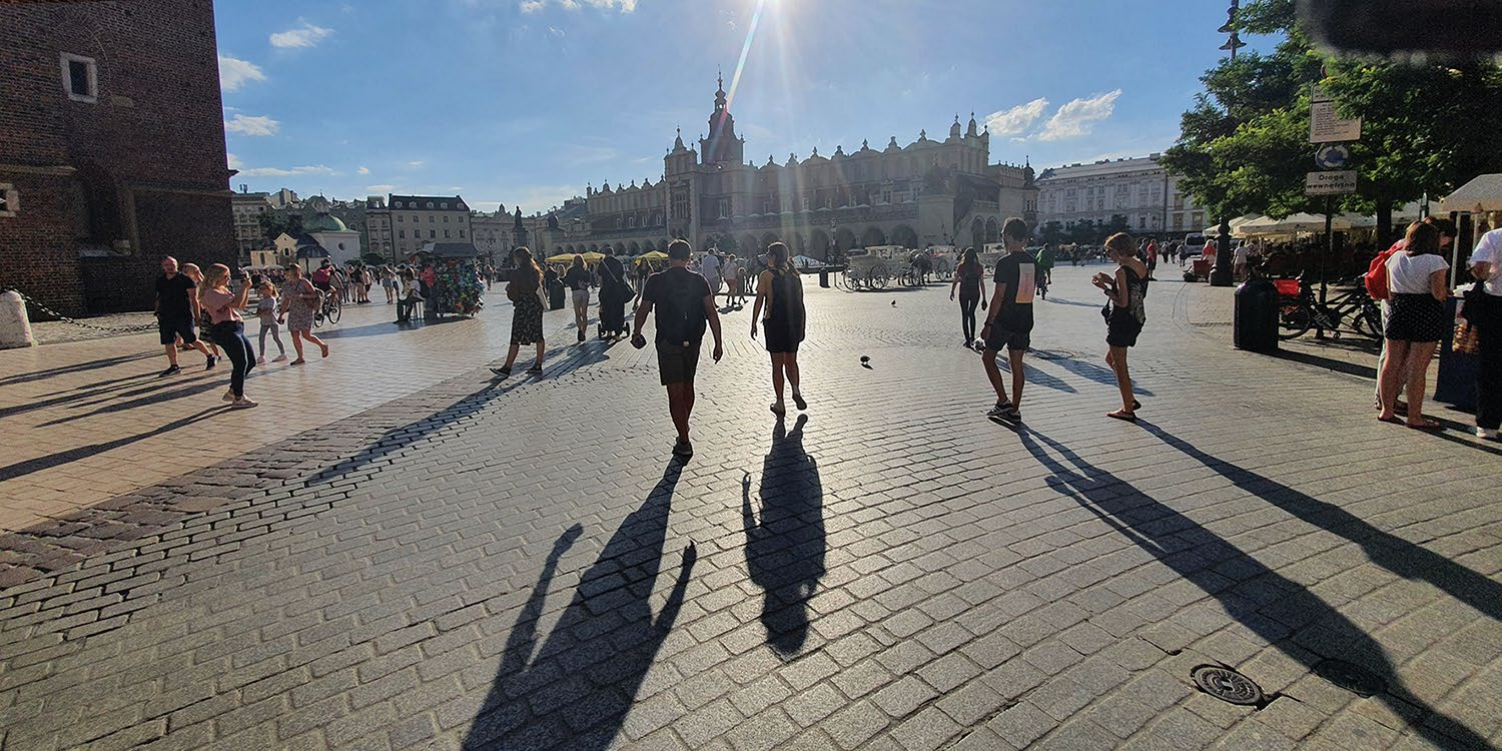
Main Square, 24 August 2020, 5 PM (62,000 confirmed cases, 548 that day). Photo by Bartosz Dendura

As for the post-pandemic period, its limits are defined not only by lifting the restrictions, but also by changes in behaviours (cf. Figs. 7, 8, 9) which at the time were not immediate (cf. Drozdowski et al., 2020a, 2020b). Despite most restrictions being lifted on 18 May, it wasn't until the milestones of 28 June, the day of the first round of presidential elections in Poland, and 1 July, when Mateusz Morawiecki, the Polish PM, declared at an event that earned media coverage nationwide that the battle against the virus was won (Watoła 2020 among others). Hence, field studies for the third phase were performed on 29 and 30 June 2020 and subsequently revized in mid-August.

#### Restriction mapping

During the lockdown, the Kraków Old Town seemed a vulnerable and costly scenography waiting for tourists to come back and requiring public support in the meantime rather than a resilient system as defined by Meerow et al. (2016) that, over the centuries, maintained its function and form. To understand the extent of the phenomenon, we recorded and mapped the spatial and functional effects of the lockdown based on onsite observation for lockdown and post-lockdown periods and reconstructed the pre-pandemic state using social media, inquiries and our personal experiences. We used a modified figure-ground diagram presenting levels of accessibility to public spaces and buildings on the ground floor levels. To measure the levels of accessibility determined by both physical and non-physical obstacles, we adopted a five-point rating scale based on the following parameters:

- level 1—no restrictions (that includes streets, squares, open courtyards before lockdown).
- level 2—mild restrictions in accessibility stemming from opening hours or soft barriers (that includes places of worship, universities, grocery stores, pharmacies, administration, etc.).
- level 3—significant restrictions in accessibility due to economic constraints or safety procedures, etc.; this category also included schools and preschools before the epidemic, as well as places that operated with sanitary regimes in place; we assumed PLN 6.88 (ca. € 1.50) as an economic barrier, as this is the amount that an average Polish pensioner can spend on culture, recreation, etc. per day as reported by the Institute of Labour and Social Affairs (these expenses are not included in minimum subsistence amounts); hence, level 3 refers to buildings with paid admittance and in which it was not possible to purchase products or services for an amount lower than or equal to the barrier value.
- level 4—highly restricted accessibility or space accessible only to a highly specific and limited group of users; this category includes, among others, residential buildings with inactive ground floors, monasteries, construction sites, etc.
- level 5—spaces and buildings that were either permanently inaccessible (e.g. abandoned buildings, buildings under threat of collapse, etc.) or made inaccessible due to restrictions.

**Fig. 10:**
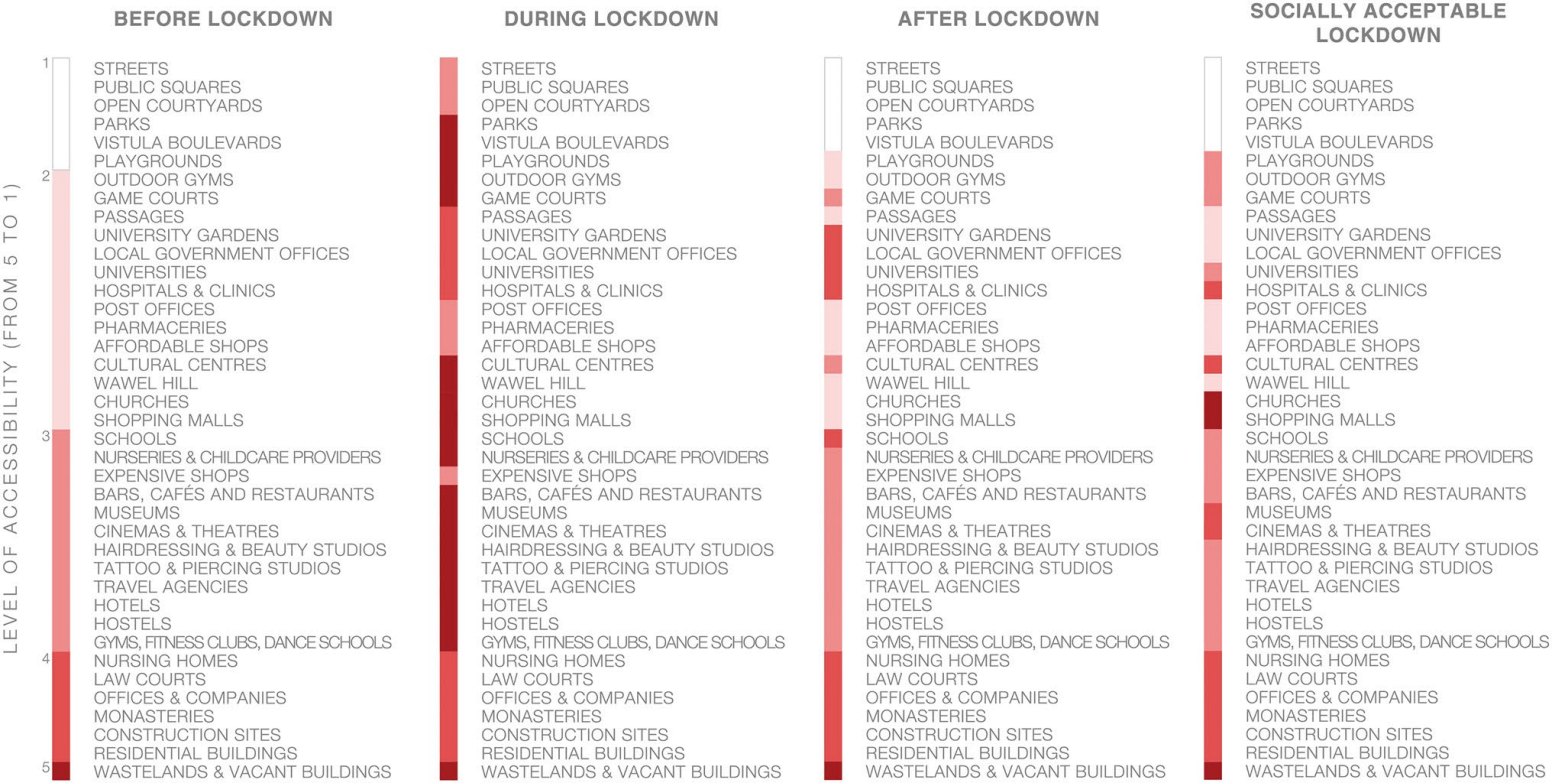
From the left: levels of accessibility before, during, and after the first wave of the 2020 Covid-19 pandemic. On the right, socially acceptable version of the restrictions as resulting from the survey

How the accessibility varied in time is summarized in Fig. 10.

The analysis of the accessibility and its impact on the structure of the city centre was limited to the ground floors for several reasons, including their undeniable role in the perception of urban spaces (Gehl 2010). We have presented accessibility levels using figure-ground diagrams so as to clearly present the findings of our analysis. The utility of the figure-ground diagram was proved by Venturi et al. (1972), Gehl and Svarre (2013) and Navarro de Pablos et al. (2019) as well as Contreras et al. (2014) who used it to demonstrate the situation of L’Aquila after the 2009 earthquake. Urban morphology, of which the figure-ground diagram is a prominent fixture, has likewise been discussed as a method of studying resilience by Danesh Pajouh and Alopouri (2019), as well as in other contexts, including public health, by Fathi et al. (2020). Accessibility studies are also a well-entrenched part of the literature (Wójtowicz-Wróbel 2017).

#### Survey

In order to include the social dimension of public spaces and places in the lockdown cartography, we conducted a survey in which respondents were encouraged to reflect on the surrounding space they were restricted to, as well as on the more distant one, which was momentarily inaccessible (Porębska and Dendura 2020). The questionnaire included 5 profiling questions (age, gender, education, place of residence by country and type of settlement) and 13 closed-ended questions concerning the lack of access to selected functions and public spaces and any impact on their use in the future, including preferences concerning transport modes. Although numerous surveys were performed during this period, they largely ignored the urban environment, focusing on behaviours within public space instead (Drozdowski et al. 2020a, 2020b among others).

**Fig. 11:**
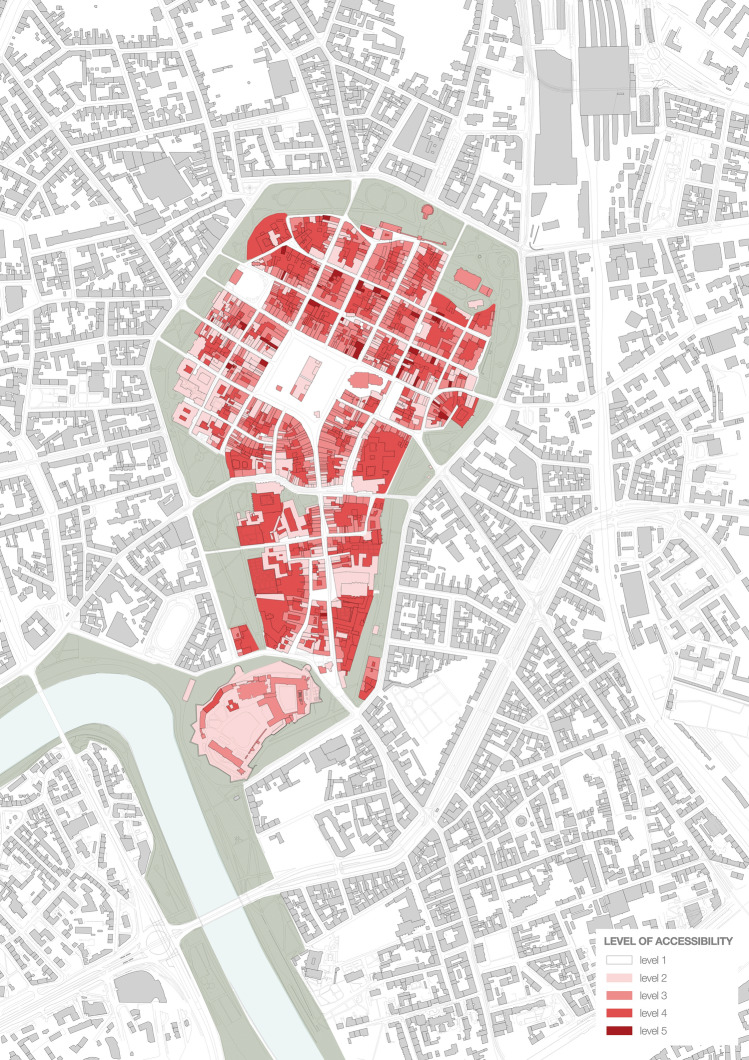
Accessibility of the ground floors and open spaces in Kraków Old Town before the 2020 Covid-19 pandemic

**Fig. 12:**
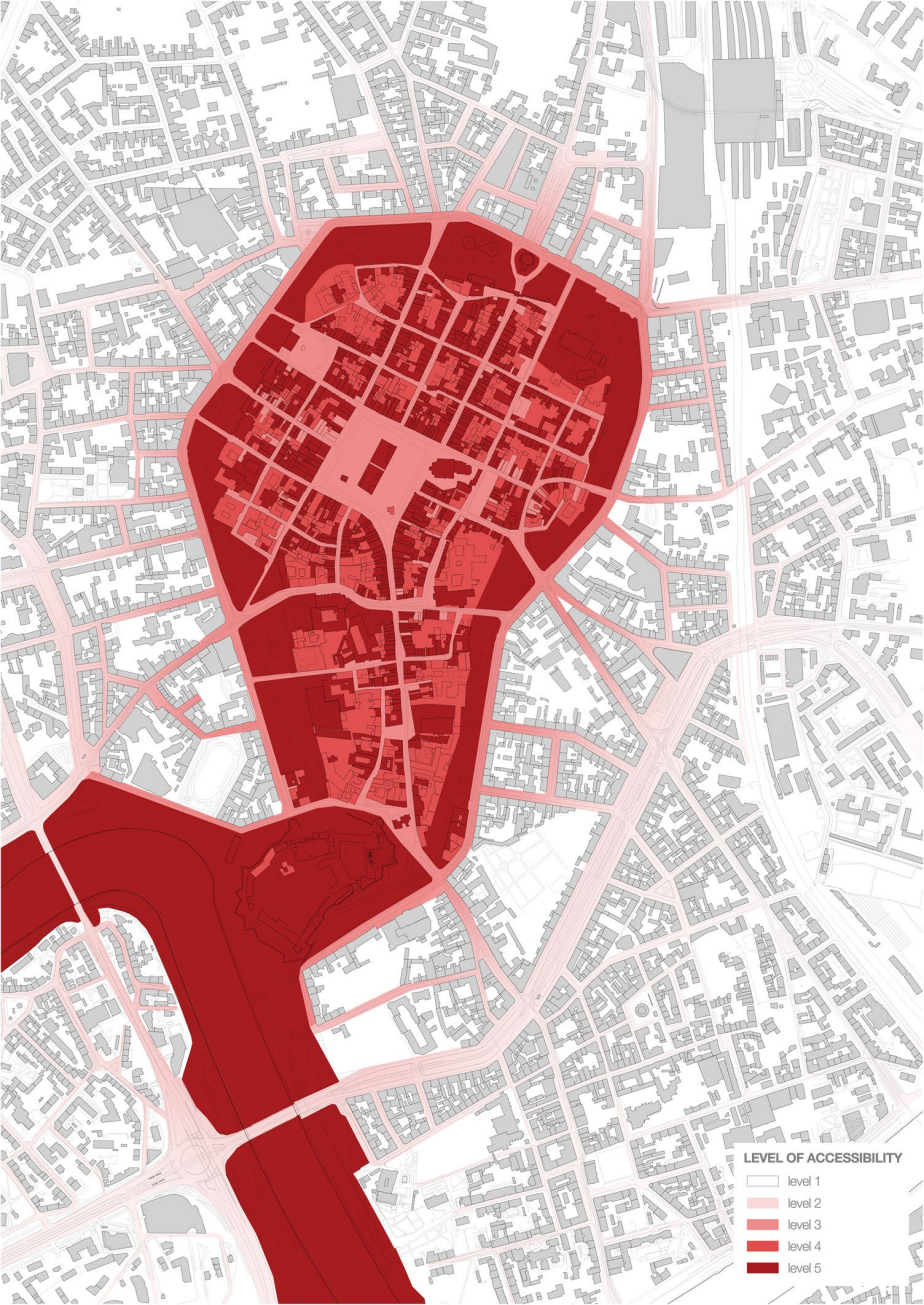
Accessibility of the ground floors and open spaces in Kraków Old Town during the first wave of the 2020 Covid-19 pandemic

**Fig. 13:**
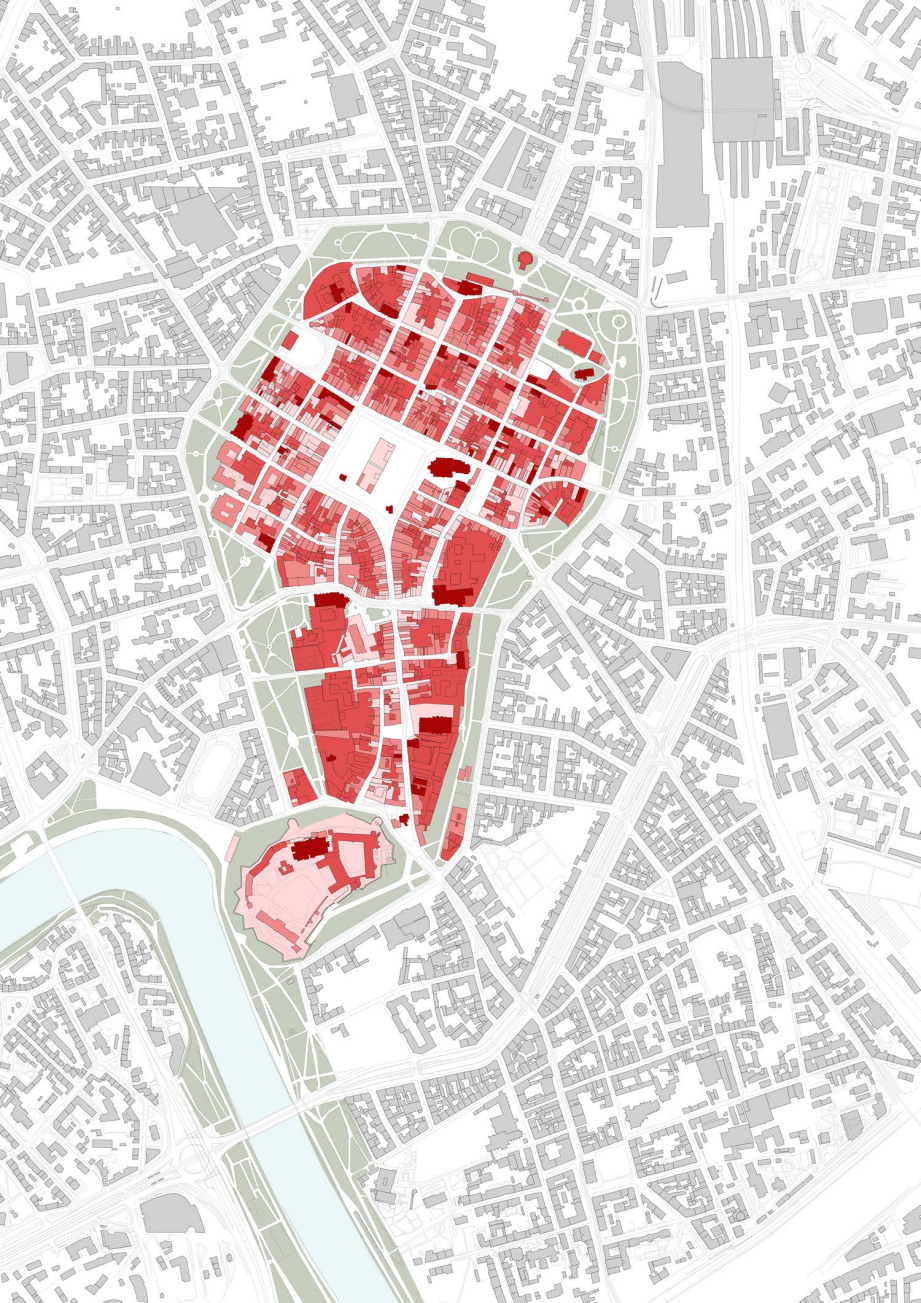
Accessibility of the ground floors and open spaces in Kraków Old Town in a socially acceptable version of the restrictions as resulting from the survey

**Fig. 14:**
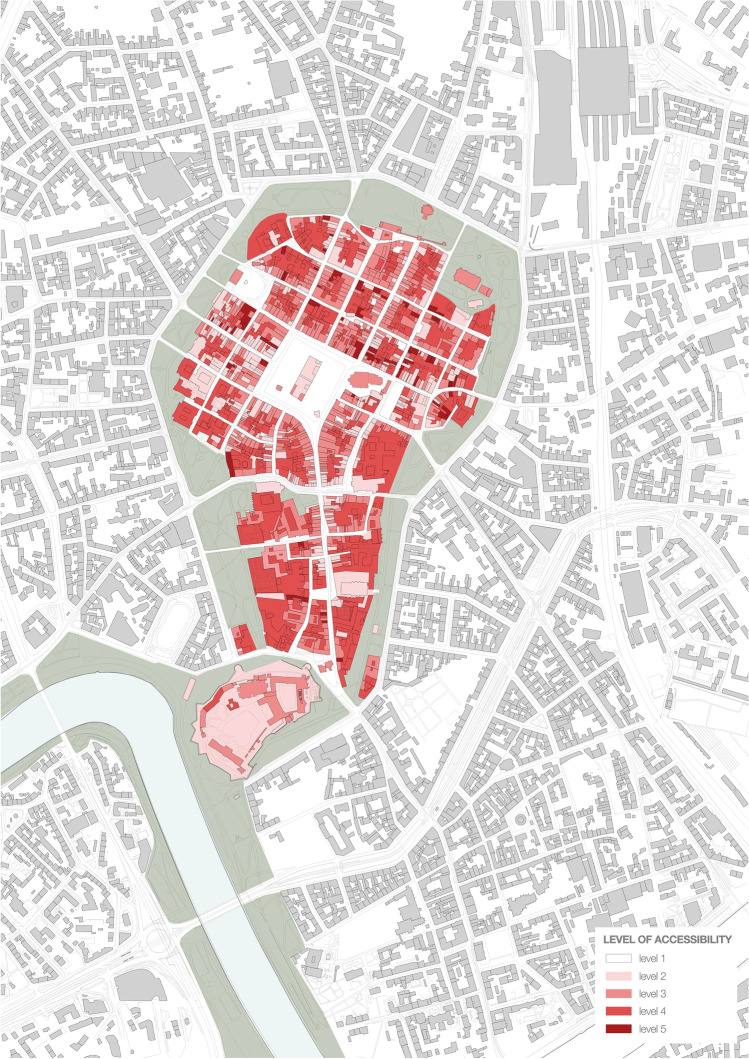
Accessibility of the ground floors and open spaces in Kraków Old Town after the first wave of the 2020 Covid-19 pandemic

The survey was performed online and was shared via a mailing list and social media, reaching over 200 respondents by 30 May. Some disproportions in gender, age, and education of the respondents were noticed. However, even if we reduced the socially acceptable lockdown model built on these findings to an illustrative material, the difference between what respondents declared acceptable and what was imposed on them would remain notable. Same as the confrontation of the deserted, temporarily suspended Old Town with the key role it plays within the entire urban system (c.f. Figs. 11, 12, 13, 14).

The results are set against the background of city’s current conservation, management and development strategies. The analysis focused on initiatives either related to the pandemic or conducted specifically to mitigate its effect among which economic recovery, mobility policies and strategies regarding green areas. The discussion is oriented towards the future of the city—as many other historical cities—which could be the one of a resilient or rather a vulnerable system.

## (Urban) space temporarily suspended: findings

### Opening hours in a disneyfied city

Before the arrival of the mass tourism that launched the process of disneyfication of the Kraków city centre, the Main Market Square played the role of an urban salon that both Jacobs (1961) and Mumford (1961) had each dreamed of in their own way. Before the pandemic, from the inhabitants’ standpoint, the greatest value of the Old Town, despite its disneyfication, were its high-quality open spaces, supplemented by accessible (level 1–2) and relatively accessible (level 3) places as presented in Fig. 11. And yet, amid the combination of the restrictions and commercialisation of the area, for several weeks during the spring of 2020, it turned into a deserted scene presented in Fig. 12.

In the culmination of the lockdown, when space and public building accessibility dropped to an absolute minimum and cycling along the Vistula was punishable with a fine of up to PLN 12,000 (close to € 3000), the Old Town became an expensive piece of scenery, largely useless even to the few who lived there. The city’s decision to close playgrounds, pitches and open-air gyms seemed justifiable at that time amid reports on high risk of transmission via surfaces. However, closing first, locally, parks and then, at the national level, also beaches, forests, and other green areas—was highly controversial instead. In the case of the Old Town, it turned Planty Park into a 40 to 120 m wide sanitary cordon. When subsequently the city decided to turn lighting off between midnight and 4 AM, it was difficult to spot a lighted window in the district.

### Socially acceptable lockdown in a disneyfied city

As most authorities worldwide, the Polish government decided to apply a heavy-handed, top-down approach in implementing restrictions. It was compliance rather than engagement that was expected of the society as if the advantages of the bottom-up approach have not been already proven in the field of disaster risk mitigation and post-disaster reconstruction (cf. Rizzi and Porębska 2020 among others). As mentioned above, the survey conducted as part of this research provided us with the material on which to speculate on a socially acceptable version of the lockdown both specifically in the area and generally, in comparison to the restrictions applied by central and local authorities. We have presented the restrictions imposed in Fig. 12, while the socially acceptable version of the lockdown is featured in Fig. 13.

Our survey was launched at a time when forests, parks, and cemeteries, along with Kraków’s Vistula boulevards, were closed. This could have had a considerable impact on the distribution in the question concerning spaces the respondents missed the most (81%—open public spaces, 19%—public spaces in buildings) and which of them should be reopened first (89% and 11%, respectively). On the other hand, the respondents’ housing conditions could have been better than average: 28% of the respondents spent this time alone, in 63% of cases every household member had their own space and only 24% of the respondents claimed that household members had to share a limited amount of space during this time (statistically, according to the official data provided by the local statistics office, the average in 2019 was 0.71 person/room).

Although the variety of remote contact tools appeared to limit the negative consequences of social isolation, respondents of the survey missed contact with acquaintances (47%) and freedom of movement (29%). Two most evident differences between Figs. 12 and 13 is the accessibility of churches vs the accessibility of open green areas. During the first wave of the pandemic, Polish government decided to keep the first open and the latter closed.

### Learning from the lockdown in a disneyfied city: findings

#### Post-pandemic economy

The cartography of the pandemic presented in the paper shows how spaces of spectacle and private sphere that since 1989 could have become those of expectation and diversion, turned into spaces of security lacking the areas of respite which, according to Carmona et al. (2019) among others, is a foretaste of decline. Setting the results of the study against official documents in this field offers a better contextualization.

Figure 14 presents the state after most restrictions were lifted and most indoor spaces could be returned to their pre-pandemic state while maintaining a less stringent sanitary regime, e.g. concerning the maximum number of users inside premises. To some, the lockdown and epidemic spelt their end. According to our field survey, from among 764 commercial premises on the ground floors only (253 shops, 171 services and 340 gastronomic establishments) that had operated in the area under study before the pandemic, 80 remained either closed or went out of business (24 shops, 26 services and 24 gastronomic establishments) as of 18 August 2020. These numbers do not include 54—mostly tourist-oriented—stalls in Sukiennice, the Renaissance Cloth Hall in the Main Market Square (Fig. 5).

Business vulnerability resulting from orientation towards external consumers and linked with the centre’s functional structure can be seen in a report on business management during the pandemic (Wysocka and Stec 2020) issued by the municipality, based on surveys from June and July. Of the business owners operating in the Old Town who participated in Wysocka and Stec’s survey, 19% declared that the pandemic had significantly contributed to their loss of financial fluidity, while in other districts this answer was given by between 0 and 4% of respondents. As many as 15% of Old Town business owners thought about completely or temporarily closing, while in other districts this amount ranged between 0 and 5%. Those out of business by mid-August 2020 correspond with approximately 10% of the ground floor commercial facilities.

Should the situation have lasted for a short period of time, the city would have probably bounced back quite swiftly. If some conclusions were drawn sooner, it would have probably bounced back stronger. Unfortunately, an official report issued by the Kraków municipality with the words *‘*pandemic-resilient city’ in its name (Barszcz 2020) focused exclusively on the role played by sectors that the pandemic hit the hardest in Kraków’s economy: the tourist, event, and airline sectors. It painted Kraków’s resilience as one largely based on ‘creating an image of Kraków as a city that is completely safe and prepared to combat the novel coronavirus both substantively and financially’ (Barszcz 2020, p. 25), with automation and AI identified as effective actions. In the light of our results, this change would have effectively been confined to marketing.

#### Post-pandemic mobility

According to the official data included in the so-called Kraków Mobility Shield, a document describing the city’s strategy regarding its mobility policy in the face of the Covid-19-related restrictions, traffic intensity in Kraków fell during the first wave of the 2020 pandemic by 30–70%. According to various estimates, the number of public transport passengers fell by 90%. Bicycle traffic was the least affected, as it fell by 50%. The bicycle became a safe alternative to public transport, one that is cheaper and more eco-friendly than a car, even in a situation when the city became an unusable barrier as seen in Fig. 12.

During the lockdown, limited public transport, the lower number of cars and considerably fewer pedestrians, enabled cyclists to travel through the centre fluidly and safely—excluding periods when travelling through Planty Park and the Vistula boulevards was punishable by a fine. However, as soon as the lifting of restrictions began, the consequences of negligence in building bicycle infrastructure became severe enough to force the city to take immediate action, including the ad hoc delineation of bicycle paths and counter flow lanes.

The Kraków Mobility Shield proposed supplementing bicycle access ways to the centre via new temporary sections and widening pedestrian spaces to enable proper social distancing. Perhaps these efforts will quicken the implementation of some elements of the 2016–2030 development strategy (Resolution of the Kraków City Council No. XCIV/2449/18 on the Kraków Development Strategy: I want to live here: Kraków 2030), which assumes increasing the share of eco-friendly mobility, including bicycles (from 4.5% in 2016 to 13–17% in 2030) but also the share of public transport (from 33% in 2016 to 38–42% in 2030) over the next decade. However, the current situation paints it as inefficient and unrealistic.

Whether the citizens are ready for changes is a separate issue. Over half of the survey’s respondents declared limiting the use of public spaces and buildings to a minimum, while not necessarily declaring restrictions on movement in itself—here the respondents were split more or less evenly. Out of all the modes of transport (car, bicycle, car sharing, bike sharing, public transport, mix, none of the above) most respondents chose their own cars. During the first week of the survey, this mode of transport was preferred by 64% of respondents, while the second-most-popular response, bicycle, was chosen by only 17%. Apart from the lifting of restrictions on access to key bicycle paths: the Vistula boulevards and Planty Park, the answers could have also been affected by a largely cold and cloudy spring (although February concluded the warmest recorded winter in Poland, 2020’s May was the coldest since 1991) and/or age. Ultimately, only 1% of respondents would choose bike sharing as a preferred mode of transport, while car sharing was not preferred by anyone.

Kraków’s bike-sharing system (Wavelo) was permanently shut down on 31 December 2019 with no replacement. The city’s streets became filled with electrical scooters, which, at that time, were just about to become regulated and are now considered equal to cyclists. The potential implementation of these regulations is another argument in favour of pro-cycling efforts, but building a new strategy based on this experience will not be easy. Especially since some of the implemented solutions are already being withdrawn as of July 2021.

#### Post-pandemic urban green areas

In the face of the Covid-19 threat, access to open spaces, especially forests and parks, continued to be perceived as critical (cf. Sharifi and Khavarian-Garmsir 2020; Cooke 2021 among others). It is a need that is difficult to satisfy in Kraków, regardless of the epidemic threat.

Ever since Ebenezer Howard called Kraków a naturally developed garden city (Bartkowicz, 2018), it has taken to promoting itself as a green city. According to studies commissioned by the local administration in 2016 (Bajorek-Zydroń and Wężyk 2016), approximately 62.3% of the city’s area is covered by some form of greenery. This also includes wasteland and private gardens. However, data published by the Polish General Statistics Office (GUS) for 2015 shows the combined share of forests, parks, greens, and housing greenery amounts to merely 9% of the city’s area, which makes Kraków Poland’s least green metropolis.

A 2017 study by the Supreme Chamber of Control (NIK) reported the amount of forest and landscape greenery per capita in Kraków to be 25 m^2^ and 24 m^2^, respectively. Should the GUS data mentioned above accounted solely for areas of the highest ecological value, namely parks (394 ha) and forests (600 ha), while subtracting street greenery (599 ha), housing greenery (854 ha), greens (310 ha) and cemeteries (138 ha), the amount of greenery suitable for effective social distancing would be 13 m^2^ per capita. Furthermore, this would hold true only for the city’s official population of 771,000, while the city’s effective pre-pandemic population, including students, tourists, commuters, etc., was estimated at 1.2 million based on water supply consumed and sewage received.

As far as the area under study is concerned, greenery occupies approximately 25% of its surface, with greenery outside of Planty Park amounting only to 10% (which is distinct for historic cities of mediaeval origin), development being 30% and the external positive space (streets, squares, etc.) 45%. It should also be stressed that out of 21 ha of Planty Park the space users have actual access to, is confined to its asphalt paths, one roofed gazebo and two fenced playgrounds. Despite walking on grass no longer being punishable by a fine, the Park’s lawns remain fenced off and those who cross them are mostly dogs and their owners. The formal, historicising character of the park not only eliminates activities that require considerable space or dedicated infrastructure, but also restricts acceptable ones, like jogging or cycling. Intensive recreation must be practised elsewhere. In the case of the UNESCO area and its buffer zone, only the Vistula River boulevards (in the south) and Błonia (in the west) are available and both areas are more than a kilometre away from the Main Market Square.

Regardless of the limited number of people dwelling the area within the area of the study, and the fact that many of the flats of the surrounding UNESCO buffer zone have been adapted to short-term lease, there are still around 11,000 people living in the proximities. Additionally, there are the residents of Kraków’s more distant areas (to whom the Old Town and Planty Park are a recreational destination or a part of their daily commute), students (the Old Town includes numerous university buildings), employees and, eventually, tourists—14 million of whom visited Kraków in 2019 according to register held by the city tourist office.

In 2019, work began on a proposal for a mixed-network system of green areas to be implemented within a decade. This proposal assumes that all residents are to have access to areas of greenery within a walkable distance (defined as 300 m or 6 min). The decision to develop the system towards a mixed direction is understandable due to the relative ease of procuring small plots of land for pocket parks and landscaping them, yet regardless of their ecological potential, such facilities can be functionally deficient in terms of sports and recreation. Only linking at least some nodal points with green pedestrian and bicycle corridors would create a zone for some forms of sports and recreation and enhance the city’s bicycle-based transport potential and residents’ well-being. Unfortunately, at present, the proposal does not feature such a strategy, while protecting ecologically valuable areas, including the third landscape as defined by Clément (2004) combined with eco-friendly mobility, should become a strategic priority for the city, both in the context of the pandemic and that of air pollution and climate change. The role the area of the study should play in building this strategy must not be underestimated as its potential is revealed if Figs. 11, 12, 13 and 14 are compared.

## Towards vulnerability or resilience? Discussion on the future of historic city centres

Since 1978, when the first UNESCO World Heritage List was published, the situation in Kraków has changed drastically in various aspects. 40 years ago, the greatest challenge for Kraków then appeared to be preserving the historic substance, which had not been renovated for decades and had been subjected to the negative impact of heavy industry sited near the city after the Second World War. Gone was the socialist regime, the fall of which did not carry over to the elimination of most of its flaws, liberal, unregulated, and often exploitative nascent capitalism mixed with conservative and sometimes erroneous conservation policies came into force.

Decades of conservative preservation policies, combined with a progressive transformation of Kraków’s Old Town into a tourist-only product, have formed a feedback loop. It generated significant profits and helped to put the city on the tourist map of Europe as a major destination during calmer times, but also sent the city on a downward spiral towards disneyification. Nevertheless, it has left the area with major vulnerabilities and placed a barrier in its path to resilience.

We displayed the effects of the Covid-19 pandemic, the associated lockdown strategies and resulting disappearance of tourist traffic which highlighted this effect painfully. As our study has shown, this disneyified and largely self-contained, monofunctional area all but collapsed in the wake of a global pandemic. It can, therefore, be concluded that disneyification, while potentially allowing for considerable economic development, should be seen as a major vulnerability and as such inadvisable as a focal point of city development strategies. Certain efforts need to be made to ensure the spaces endangered by it more liveable and to retain as many permanent residents and housing areas as possible.

While efforts are made to combat the Covid-19 pandemic with lockdowns and vaccinations, there is currently no end to it in sight as new, potentially even more dangerous strains of the virus are emerging. Further research into the behaviour of the built environment during a pandemic and its long-term effects on disneyified spaces appears to be a promising field. One avenue that can be pursued is the ‘behaviour’ of spaces located on the edges of a disneyified core area. They can potentially be affected by this negative phenomenon, but they can also create a link positively acting between inclusive, healthy spaces and embellished, artificial tissue.

The pandemic had highlighted the health inequalities due to insufficient access to green space and poorly designed homes and places which has rightfully put these issues in the centre of the recovery plans. After the UK government had signalled its intent to outline proposals for comprehensive reform to England’s planning system, planners and local stakeholders expressed their doubts whether now was the time to destabilize planning systems rather than strengthen them (cf. Local Planning Authorities 2020). In the UK and the EU (cf. Recovery Plan for Europe 2021), declarations are being made regarding a better, greener, more sustainable and more resilient future, but deregulations are still being seen as key to delivering on those promises. The recovery plan for the UK unveiled during the ceremonial opening of the new session of Parliament on 11 May 2021, is expected to be the most radical since the 1948 Town and Country Planning Act, and not in a positive way (cf. Booth 2021, among others). The aim is to build a lot more and to build faster, but not better, and the simplification of environmental assessments for developments does not seem to be oriented towards ensuring access to high-quality green areas or reducing the carbon footprint of the projects. The same is the case of Poland (cf. Polish National Recovery Plan 2021), where forests can now be turned into construction sites and houses with a floor area of up to 70 m^2^ are supposed to be built without building permits, topped only by the decision on coal mines being kept operational until 2050. As proven by the example of Kraków and its historic city centre presented above, there is no such thing as conservative deregulation. And a future built on rapid and unregulated development is a vulnerable and not a resilient one.

## Conclusions

In our paper, we have presented how, during the Covid-19 pandemic, the historic city centre of Kraków, Poland, evolved in terms of accessibility. From a bustling, hyperactive tourist destination all but turned into a historically authentic theme park, it became a ghost town in the wake of government-imposed restrictions. This laid bare decades of deficiencies, paradoxes and poor decisions made after the fall of communism in Poland, clearly demonstrating a need for smarter and more resilience-oriented planning and land-use regulations that would holistically encompass architectural conservation, building use regulations, mobility and urban greenery. As the Covid-19 pandemic is not yet over at the time of writing of this paper and the situation in Kraków continues to change in a more balanced approach to tackling the spread of the novel coronavirus, we believe that further monitoring of the events that unfold will bring considerable insight into urban resilience during this crisis.
